# Supplementary material for: The hidden cost of a smartphone: The effects of smartphone notifications on cognitive control from a behavioral and electrophysiological perspective
Source: PLoS One. 2022 Nov 17;17(11):e0277220. doi: 10.1371/journal.pone.0277220 (PMC9671478; doi:10.1371/journal.pone.0277220)
Supplement: Attachment — Submitted filename: Response to Reviewers.docx [file pone.0277220.s002.docx]

Dr. Francesco Di Russo	October 17, 2022

*PLOS ONE*

Dear Dr. Di Russo,

Thank you for your consideration of our manuscript entitled “The hidden cost of a smartphone: The effects of smartphone notifications on cognitive control from a behavioral and electrophysiological perspective.” for publication consideration with *PLOS ONE*. We have. addressed the helpful reviews and made changes according to the reviewer’s suggestions (in blue font in the manuscript). We detail these changes below.

**Editor and Journal Requirements** .

When submitting your revision, we need you to address these additional requirements.

1. Please review your reference list to ensure that it is complete and correct. If you have cited papers that have been retracted, please include the rationale for doing so in the manuscript text, or remove these references and replace them with relevant current references. Any changes to the reference list should be mentioned in the rebuttal letter that accompanies your revised manuscript. If you need to cite a retracted article, indicate the article’s retracted status in the References list and also include a citation and full reference for the retraction notice.

**Author Response:**

We have double checked all articles to ensure that they have not been retracted. We employed Zotero reference manager and google scholar for retraction checks.

**Reviewer Comments to the Author**

Reviewer #1: The authors have responded to all of my previous comments to my satisfaction. My only concern is that the Github repo is still private, so I had not had a chance to fully examine the LMER model and make sure I understand them correctly. Per PLOSONE policy, the repository MUST be made public before publication (I would also recommend sharing it through OSF).

**Author Response:**

We have created a publicly available OSF repository that includes the R scripts and data files to improve transparency. This can be accessed here: <https://osf.io/bj7zf/>

The data availability statement will also be adjusted accordingly.

**MINOR comments**:

1. “An intraclass correlation of r = 0.14”. I noted this in my previous review, I am not sure if the authors actually mean ICC (such as ICC = 0.14) or Pearson correlation.

**Author Response:**

Thank you for catching this typo. We have adjusted the sentence appropriately.

“An intraclass correlation (ICC) of 0.14 for RT within participant was found, warranting the use of mixed linear models.” (p. 15)

2. The participant info (number, age, proportion female) is repeated twice, in the special Participant subsection and then again below.

**Author Response:**

We have removed the unnecessary participant info as it did not provide additional useful information.

“Four participants were excluded from behavioral data analyses due to technical issues or poor performance on the oddball task (i.e., errors or RTs exceeding +/- 2.5 *SD*). The final sample for behavioral analyses included 69 participants. ERP data for 19 participants were excluded because of technical issues, or for having uncorrectable artifacts greater than 25% of total trials [48]. The final sample for ERP analyses included 54 participants.” (p. 12)

3. Page 15 “ This model was found to provide the best fit of the data.” Best compared to… What? Maybe other, simpler LMER models?

**Author Response:**

Thank you for pointing out this area of confusion. We have adjusted the analytical strategy section to improve the clarity on these points.

“For RTs, analyses were conducted using linear mixed effect regression (LMER) models with random slopes for the trial frequency condition (rare and frequent) and random intercepts for each participant to account for within-subject variance in RT across all trials (lmerTest v. 3.1.1) [57]. This model was found to provide the best fit of the data relative to simpler LMER models.” (p. 15)

4. Page 16. After explaining the logistic regression model for accuracy, the authors note that “ Error rates were assessed using the same predictor variables .”. But… Aren’t error rates just the complement of accuracies? And doesn’t the logistic model already provide a complete account?

**Author Response:**

Thank you for pointing out this redundant information. We have adjusted the paragraph to improve clarity.

“For the accuracy analyses, we conducted generalized linear mixed effects regression model with error rate as the dependent variable, random slopes for trial frequency, and random intercepts for participant. We used a bound optimization by quadratic approximation with a binomial family distribution of 0 for correct and 1 for incorrect trials.” (p. 16)
